# Learning Away from Home. A Qualitative Study of General Practice Trainees’ Educational Experiences in Hospitals

**DOI:** 10.5334/pme.1981

**Published:** 2026-03-30

**Authors:** Tine Lass Klitgaard, Jane Ege Møller, Signe Gjedde Brøndt, Flemming Randsbæk, Søren Prins, Trine Lignell Guldberg, Joachim Frølund Hansen

**Affiliations:** 1 Department of Clinical Medicine, Aarhus University, Denmark; 2 Department of Postgraduate Medical Education, Aalborg University Hospital, Denmark; 3 Department of Clinical Medicine, Aalborg University, Denmark

## Abstract

**Introduction:**

During general practice (GP) specialist training, trainees typically complete several hospital rotations to develop specific skills and manage a wide range of medical conditions. Previous research indicates that such rotations can also present challenges, including feelings of isolation and risk of burnout. However, little is known about how GP trainees themselves experience and navigate their learning in hospital settings. Understanding these perspectives is essential for designing supportive postgraduate training environments. This study therefore explored GP trainees’ experiences of hospital rotations in Denmark.

**Methods:**

We conducted a qualitative study combining 38 hours of field observations following nine different GP trainees and ten group interviews with a total of 44 participants. Data were analysed using principles of reflexive thematic analysis.

**Results:**

GP trainees regarded hospital rotations as essential for developing competencies needed in general practice, yet they also described them as challenging to navigate. Through our analysis, we developed three overarching themes: 1) Finding meaning away from home; 2) Learning in frequent transitions; and 3) Working and learning at the margins.

**Discussion:**

Hospital rotations provide valuable learning opportunities, but their educational impact is shaped by how relevance is made explicit and how transitions are supported. For GP trainees, frequent hospital rotations combined with time away from general practice can create a form of double peripherality, making continuity and belonging fragile. Clear framing of learning objectives, structured rotation planning, and accessible peer and supervisor support are key to ensuring educational value and fostering continuity, belonging, and the development of GP professional identity.

## Introduction

Throughout the world, specialist training typically involves a series of rotations in which junior doctors work across various clinical settings to develop both specialised skills and broader competencies. These rotations may take place within units or departments of the trainees’ own specialty, but they can also include related specialties outside their primary field. While designed to enhance general clinical capability, research has increasingly drawn attention to how such rotational structures not only shape learning opportunities, but also trainees’ professional development and the educational and professional challenges they may introduce [1,2,3].

General practice (GP) specialty training is one such programme characterised by frequent transitions between clinical settings. This training often includes multiple hospital-based rotations to develop pattern recognition and competence in managing common medical conditions in high-prevalence settings [4,5], and, as a result a substantial portion of their training takes place outside primary care [1]. However, a recent review found that the duration, number, and integration of hospital rotations in GP training varies considerably across countries [3].

Existing research highlights several interrelated tensions associated with GP trainees’ hospital rotations. First, studies indicate that some trainees may struggle to find meaning in hospital rotations, since their primary focus lies in general practice [1,6]. Research further suggests that formal teaching and assessments in hospitals can be poorly aligned with the competencies required in general practice [1,2,6]. Together, these findings point to challenges in how relevance and purpose are established across training contexts.

Second, research documents high workloads, time pressures and rising stress levels among GP trainees, including early signs of burnout [7,8]. Such conditions have been linked to risks for patient safety and quality of care [9], and are associated with increased sick leave and intentions to leave the profession across specialties, as shown in a recent review [10]. This body of work underscores the importance of attending to GP trainees’ working and learning conditions during hospital rotations.

Finally, beyond issues of relevance and workload, hospital rotations are also embedded in structures that shape trainees’ learning opportunities. Studies highlight how professional hierarchies and cultural power dynamics shape GP trainees’ participation and sense of legitimacy within hospital teams [11,12]. Many GP trainees describe experiences of isolation, reduced status or being positioned as “outsiders” within hospital settings [1,2,11,13]. These findings illustrate how learning during hospital rotations is closely intertwined with social relations, organisational structures, and opportunities for participation.

Despite this growing body of work, fewer studies have focused specifically on how GP trainees themselves make sense of their hospital experiences – what they find meaningful, challenging, or valuable for their professional development across clinical settings characterised by frequent transitions. Attending to these perspectives is crucial for shaping training environments that support learning, identity formation, well-being, and ultimately the quality of care.

This study explores how GP trainees experience their specialist training in hospital settings, and what opportunities and challenges these rotations present from their perspective as part of the existing training structure. In this context, the Danish GP training programme represents a model in which hospital rotations constitute a relatively extensive part of the overall training and involve placements across multiple medical specialties, making transitions between contexts a defining feature of the training experience.

## Context

After completing their medical degree, Danish doctors undertake a one-year clinical internship comprising two six-month rotations in hospital departments and general practice. This is followed by a six-month introductory residency position, which qualifies them to enter general practice specialist (GP) training.

**Figure 1:**
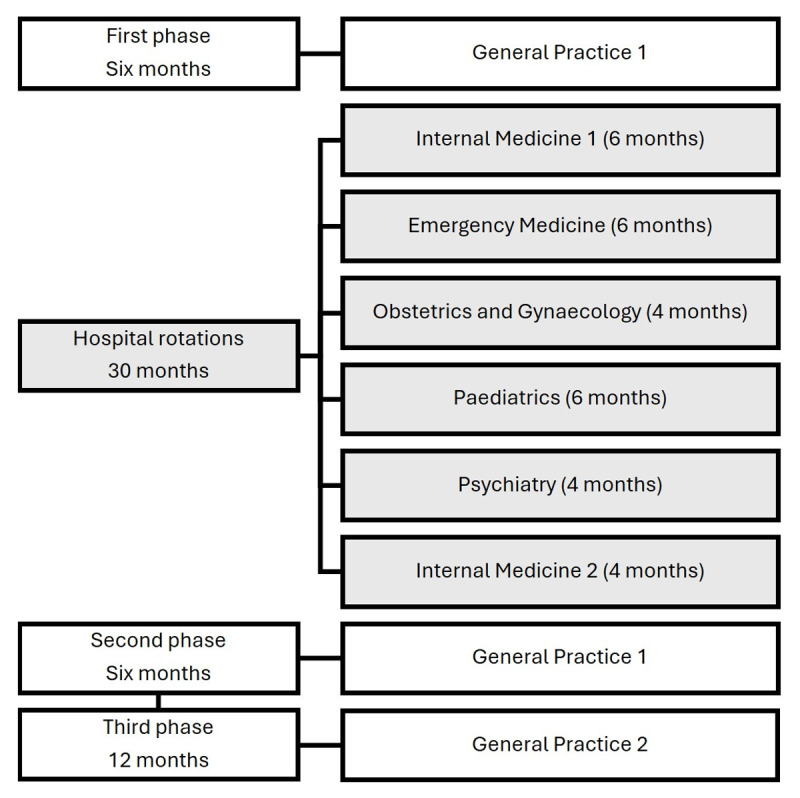
Danish General Practice Specialist Training Programme. The figure illustrates the overall organisation of the general practice (GP) specialist training, comprising a predefined sequence of four major phases. While the duration of these overarching phases is fixed, the specific configuration of hospital rotations including their length, order, and departmental composition varies between programmes. This example represents a typical, but not prescriptive, trajectory through the programme. The shaded segments indicate the hospital-based rotations that constitute the focus of the present study.

GP specialist training in Denmark spans five years (including the introductory residency position), with approximately half of this time spent in hospital departments (see Figure 1 for an overview). The first phase includes six months in a GP practice, followed by two and a half years of hospital rotations. These hospital rotations typically include departments such as internal medicine, obstetrics and gynaecology, paediatrics, psychiatry, and emergency medicine, each lasting between three and nine months.

Typically, around four GP trainees start their hospital rotations at the same time, although departments often host GP trainees from several rotation cycles, resulting in between five and ten GP trainees working in the same department at any given time. During these hospital rotations, GP trainees work alongside hospital-based specialty trainees and perform clinical duties equivalent to those of other junior doctors within the departments to which they are assigned. To support continuity and integration of learning, GP trainees also return to their first-phase GP practice approximately one day per month (“return days”). The final two phases consist of two further GP placements of six and twelve months, respectively [14].

## Materials and Methods

### Study design

To explore GP trainees’ experiences of hospital rotations, we conducted a qualitative study incorporating observations and group interviews. Combining these methods enabled us to explore both organisation, collaborations, and personal experiences. While the group interviews captured GP trainees’ retrospective reflections on their work, the observations provided insight into their practices and interactions within the clinical environment.

### Data generation and participants

Participants were recruited from among GP trainees in the Central Denmark Region and the North Denmark Region. All GP trainees in these regions currently undertaking hospital rotations were informed about the study by email and invited to contact the research team if they wished to participate.

The field observations were planned in collaboration with the department management, aiming to include GP trainees from different hospital departments and specialties. Prior to each observation, all doctors were informed by email and invited to contact the research team with questions or to opt out. Departments were also asked to inform other healthcare staff about the planned observation days. On the day of observation, the project was reintroduced at the morning report, emphasising that participation was voluntary. Several GP trainees chose to take part in each observation.

The field observations comprised 38 hours of observation across five hospital departments, with each lasting approximately 7–8 hours. During these visits, one of the researchers (TLK) followed nine GP trainees in their daily clinical work to capture a broad range of experiences (Table 1), spanning activities such as on-call shifts, outpatient clinics, and ward rounds. During the observations, field notes were written [15]. The observations also informed the development of the group interview guide. When observations involved patients, both patients and staff were informed about the researchers’ presence and provided verbal consent.

**Table 1:** Participant information.


FIELD OBSERVATIONS		GROUP INTERVIEWS	
	
GP trainees		GP trainees	
	
Male	4	Male	13
	
Female	5	Female	31
	
Speciality		Speciality	
	
Internal Medicine	5	Internal Medicine	19
	
Psychiatry		Psychiatry	10
	
Paediatrics	2	Paediatrics	1
	
Gynaecology		Gynaecology	1
	
Emergency medicine	2	Emergency medicine	13
	
Participants in total	9	Participants in total	44


In total, ten group interviews were conducted with 44 GP trainees (3–6 participants per group) (Table 1). Group interviews typically took place during work hours and often within the same department where GP trainees were based. Each group interview lasted 45–90 minutes, was audio-recorded, and subsequently transcribed. The group interviews followed a semi-structured format, using a guide to ensure coverage of key themes while allowing participants to elaborate freely [16,17]. The interview guide was iteratively refined during data collection, incorporating insights from concurrent field observations, for example, when issues concerning the visibility of GP trainees’ competencies emerged. An English translation of the interview guide is provided in Appendix 1.

### Analysis

Our study is informed by a constructivist epistemology, which assumes that knowledge is produced through social interaction and shaped by context, language, and interpretation [18]. Qualitative research follows a circular process in which data collection, analysis, and writing are closely interwoven [16]. Our analysis was guided by principles of reflexive thematic analysis [19,20], a methodology that emphasises deep engagement with the data through an iterative process including reading, reflecting, questioning, writing, and returning to the material [19]. An overview of our analytical process is presented in Table 2.

**Table 2:** The analytical process.


PHASE	ACTION	DESCRIPTION

1.	Familiarisation; reading and initial coding of group interviews	All authors independently read and initially coded the same three group interview transcripts, noting potential areas of analytical interest. At the first meeting, group interviews were reviewed paragraph by paragraph, and early interpretations were discussed.

2.	Development of codes and themes	Five additional group interview transcripts were read, building on the preliminary areas of interest identified earlier. These were organised into broader conceptual categories, forming a preliminary coding framework. In addition, two of the authors (TLK and JEM) analysed the field notes to incorporate observational data. The analysis of categories continued until consensus was reached on the final themes and coding framework.

3.	Final coding	Two authors (SP and FR) read all group interview transcripts in their entirety to ensure a comprehensive understanding of the material. Subsequently, the five last authors individually coded the transcripts. This process resulted in a shared coded dataset, which served as the foundation for drafting the results section.

4.	Writing	All authors took part in writing the final analysis.


### Research team and reflexivity

The research team comprised members with diverse professional and disciplinary backgrounds: Anthropology (TLK), scholar of literature, communication and philosophy (JEM), general medicine (SGB, SP, and JF), and hospital-based physicians (FR and TLG). All doctors had responsibilities for postgraduate medical training. This composition brought together both outsider perspectives, as non-clinicians provided an outsider perspective that helped to surface and question implicit assumptions about hospital culture, and insider perspectives as the GP and hospital-based members contributed insights into clinical work and the organisation of training. Throughout the research process, reflexive discussions were used to make these positionalities explicit and to consider how they might influence interpretation, thereby balancing contextual familiarity with analytical distance [21].

### Ethics and Consent

This study was conducted in accordance with the ethical principles outlined in the Declaration of Helsinki for medical research involving human participants [22]. The regional ethics committee reviewed the project and declared that formal ethics approval was not required (2024-000488). The study was registered with the Data Protection Agency at Aarhus University.

Throughout observations and group interviews, the participating doctors were informed about the project, and written informed consent was obtained prior to both observations and group interviews. All quotes and identifiable details were pseudonymised. In this article, each participant is referred to by both the group interview number and a unique participant ID (e.g. *Group interview 3, GP trainee 4*), where the ID reflects the participant’s numerical order in the study.

## Results

Our analysis highlighted how the GP trainees perceived the hospital rotations as essential for acquiring the skills and competencies required in general practice, while at the same time experiencing them as quite challenging and, at times, overwhelming:

Well, you really learn an incredible amount professionally. Honestly, you learn so much. It’s just in a really tough way. So, it’s something that you just have to get through, but once it’s over, it’s really good. At least that’s what I tell myself. (Group interview 2, GP trainee 8)

Across the group interviews, GP trainees expressed varied and sometimes ambivalent perspectives about the structure and purpose of hospital rotations, including their ideal duration, the choice of specialty, and work tasks. The following sections present how these complexities were analytically organised into three overarching themes: 1) Finding meaning away from home; 2) Learning in frequent transitions; and 3) Working and learning at the margins (Table 3).

**Table 3:** Results.


THEME	SUB-THEMES	BRIEF DESCRIPTION OF THE THEMES

Finding meaning away from home	Experiencing distance from general practice	GP trainees described how hospital rotations created a sense of distance from “home” in general practice, prompting frustration about lost continuity as well as deliberate efforts to maintain a GP perspective.

	The relevance of hospital rotations	GP trainees expressed ambivalence regarding the relevance, describing both valuable foundational learning for future general practice and frustration with tasks perceived as poorly aligned with their anticipated role.

	Contextualising relevance through supervision and objectives	The relevance was shaped by supervisors’ ability to contextualise learning and by the alignment of learning objectives and activities with general practice needs.

Learning in frequent transitions	Navigating new environments	Repeated transitions were experienced as demanding, with time and energy initially devoted to navigating local systems rather than to professional learning.

	Adapting to new roles and responsibilities	Frequent transitions required continual adjustment to shifting roles, responsibilities, and hierarchical positions, making it difficult to establish professional footing and a sense of belonging.

	Seeking continuity and belonging through GP peer relations	Relationships with GP peers provided continuity, mutual support, and a sense of belonging that helped GP trainees navigate frequent transitions.

Working and learning at the margins	Fitting in and having a place	Learning and well-being were shaped by the extent to which GP trainees were recognised, included, and given a place within hospital teams, with belonging requiring active effort rather than being taken for granted.

	The challenge of balancing educational needs and service demands	High service demands often took precedence over educational needs, making it difficult to balance learning with clinical workload.


### Finding meaning away from home

The first theme describes how GP trainees perceived their hospital rotations as both intense and educational, yet also distant from their professional ‘home’ in general practice. We identified three interrelated aspects: 1) Experiencing distance from general practice; 2) The relevance of hospital rotations; and 3) Contextualising relevance through supervision and objectives.

#### Experiencing distance from general practice

GP trainees expressed a wide range of experiences. Some viewed hospital rotations as a “fantastic opportunity” to encounter a “concentration of relevant patients” and to learn from hospital specialists. Others, however, expressed frustration about spending much of their training in hospitals – settings they had deliberately left behind when choosing general practice:

I chose a specialty like general practice. So, for me, at least, it was also about wanting to get away from the hospital. I don’t really like that workplace. (Group interview 5, GP trainee 19)

For some, this return to hospital felt like being “forced into it anyway”, highlighting tensions between specialty choice and organisation of training. The sense of distance they experienced was not only geographical but also professional:

What I feel you lose a bit, is that GP perspective. It’s like, just when you were starting to feel like you could see through those lenses […]. So, I kind of feel like you have to start all over again when we’re done with the hospital rotations. We’ve gained a lot of knowledge, yes. And we can use it, which is really great. But you lose that, well, the GP aspect of it. And I think that’s something about our education. I don’t quite understand why it’s like that. […] But it’s a bit of an odd education. In the sense where I feel like we end up quite far removed from our own specialty. (Group interview 4, GP trainee 15)

Here, the “GP perspective” is presented as something requiring time and continuity to cultivate, and the comment “you have to start all over again” suggests that the hospital rotations are perceived not just as pauses, but as setbacks.

Yet, through our analysis we identified a contrasting dynamic. Although GP trainees described feeling distant from general practice, some actively worked to preserve it. They did so by using GP-specific reference materials or by defending general practice when it was criticised by hospital colleagues. These efforts illustrate how they sought to orient themselves towards general practice despite being embedded in a hospital environment.

#### The relevance of hospital rotations

Across both observations and group interviews, GP trainees expressed ambivalence about the relevance of hospital training, ranging from finding it highly beneficial to questioning its applicability to general practice. Many valued gaining experience with initiating treatments, gaining insight into patients’ treatments, practicing watchful waiting, and learning to judge when to manage patients in general practice versus to refer them:

I now know what the patients are going through. I actually have a sense of what I’m sending them into. When I refer a patient for a specific evaluation or procedure, I know what’s going to happen. That makes a huge difference because patients ask questions, and you wouldn’t know if you didn’t have that insight. (Group interview 8, GP trainee 34)

These insights were often described as “foundational knowledge” for future GP work. However, perceptions of relevance varied: what some considered highly relevant, others viewed as largely irrelevant or detached from their future role.

At the same time, many GP trainees were frustrated by tasks perceived as having limited relevance to general practice such as covering a highly specialised outpatient clinic, performing procedures like electroconvulsive therapy, or ventilating a newborn. Such mismatches between tasks and perceived learning needs led to dissatisfaction:

I think it’s true that you obviously gain broad knowledge. But there are still many things that you can’t relate to your work in practice. Because it’s just a completely different setup here […] For example, when you have a very complicated patient, where you have all sorts of specialists involved. You quickly reach your limits when you’re working in a general practice. And that’s where I think it becomes hard to figure out what exactly I will need to use. (Group interview 4, GP trainee 14)

#### Contextualising relevance through supervision and objectives

While many GP trainees encountered tasks they felt were of limited relevance, others described how supervisors could enhance meaning by explicitly connecting activities to general practice learning goals. Departments considered most relevant were those that planned and organised learning opportunities with GP needs in mind; outpatient clinics and ward rounds were highlighted as particularly valuable:

In [one specialty department], they also have separate outpatient clinics […] for the GP trainees […] tailored to what we need to learn, rather than just being solely about contributing to the workload. […] it’s just so great when it’s relevant to us. (Group interview 8, GP trainee 30)

Some participants were surprised more effort was not made to organise training in this way, though they recognised that even well-intentioned efforts could be undermined by service pressures:

I also think the department’s intention is for it to be relevant. But then you run into illness, and then you run into production. And then you end up in a specialist outpatient clinic… (Group interview 5, GP trainee 19)

Hospital supervisors played a central role in contextualising learning, and accessible supervision with an understanding of general practice was seen as essential to ensuring relevance. This was not always the case:

My supervisors, I don’t think they really know what I have to go through. […] They always ask, “what exactly are you supposed to do”? (Group interview 7, GP trainee 26)

GP trainees acknowledged that this lack of alignment also posed challenges for the supervisors themselves, who were often far removed from the realities of general practice and therefore uncertain about the competencies GP trainees were expected to develop:

I think sometimes it’s also difficult for the specialists. They’re so far from what everyday life is like in general practice, so I don’t think they always know what kinds of competencies we actually need. (Group interview 4, GP trainee 16)

GP trainees further noted that the GP training framework offered limited guidance to hospital departments, describing them as “loose” and non-prescriptive. As such, relevance was not only an individual concern but also a structural and organisational challenge. Without attention to how learning opportunities are organised and adequately prepared supervisors, efforts to ensure relevant learning risks becoming fragmented and dependent on local initiative.

### Learning in frequent transitions

The second theme captures how GP trainees experienced the organisation of hospital training as a series of short rotations. Although views differed on the ideal rotation, many described the frequent transitions as a major challenge: “really tough”, “terrible”, “exhausting”, and “something to get over with”:

Mentally […] it impacts you, so you become both transition-weary and relationship-weary. You spend the first month -or more -in each department just feeling completely overwhelmed. You can hardly take anything in. At least, that’s how I feel. And then, when you just about reach the surface […] you start to feel some calm in your daily work and your professional development. […] And then you start all over again. (Group interview 2, GP trainee 8)

The following sections illustrate how GP trainees perceived these frequent transitions through three interrelated aspects: 1) Navigating new environments; 2) Adapting to shifting roles and responsibilities; and 3) Seeking continuity and belonging through GP peer relations.

#### Navigating new environments

GP trainees most often linked the difficulty of transitions to the need to repeatedly learn and adapt to new clinical environments. Each rotation required them to familiarise themselves with different systems, hierarchies, and social norms, often with limited time for structured introduction or gradual immersion. The experience of continually “starting over” was both practically demanding and mentally exhausting:

On the first day, you always think, like… “I’ll never learn to find my way here”. (Group interview 2, GP trainee 9)

On the second day, I walked in [to my colleagues], “What’s your name? You’re getting a patient in room eight”. “Where is room eight?”. Like, “What am I supposed to do?”, “Who should I talk to?”. So it was just… chaos. […] It would have been nice with a bit more structured [introduction]. (Group interview 3, GP trainee 12)

These accounts illustrate how the initial period of each rotation was dominated less by clinical learning than by navigating local logistics, such as finding one’s way, understanding local routines, and learning administrative procedures:

In the beginning, a lot of time is spent figuring out the practicalities. Where do I park? Where is my locker? How do I attend the morning conference? Where do I report absences? What about overtime? And in this outpatient clinic, should I dictate or write it myself? Should I send it to the secretary, or do they want a short note? I spend a lot of energy navigating all of this, so it simply takes more time before I can start to benefit professionally. (Group interview 9, GP trainee 37)

While such logistical challenges are familiar to any doctor entering a new department, GP trainees faced them repeatedly across different specialties and hospital systems. What might be an occasional disruption for other trainees became a recurring feature of their training. As a result, the first weeks of each rotation were often perceived as a pause in learning, a period of survival and adaptation rather than professional growth. This constant resetting of knowledge and relationships contributed to the feeling, as one participant put it, of being “forever new”.

#### Adapting to new roles and responsibilities

Beyond navigating new environments, GP trainees described the challenge of continually adjusting to shifting roles and responsibilities. Moving between departments meant encountering new hierarchies, clinical norms, and responsibilities. Several described the experience as confusing or even disorienting, particularly when expectations differed markedly between departments, either unrecognised as competent doctors or expected to take on more than they felt able to or that they should deliver:

And you have to have a lot of antennas up. There’s an enormous amount of adaptation. For example, going from the emergency department, where you work as a middle-ranking resident, you’re relatively high in the hierarchy and in seniority […] And then you move to [another department], where you’re clearly the lowest in the hierarchy and seniority and are the one who doesn’t know anything. So, finding your place—where is my place in all this? (Group interview 2, GP trainee 9)

These frequent transitions made it difficult to establish a stable professional footing, as each rotation brought new expectations and power dynamics with little time to consolidate learning or to build trust within new teams. Several trainees described feeling alternately under-recognised and over-exposed. They highlighted the need for structured introductions, not only to ease the transition but also to support psychological safety and a sense of belonging.

#### Seeking continuity and belonging through GP peer relations

As they navigated new departments and shifting roles, GP trainees often turned to fellow GP peers for support and reassurance. Across the group interviews, these relationships were described as a crucial source of community. For many, being placed alongside the same group of GP trainees during the hospital rotations provided a sense of ‘team spirit’ and familiarity in otherwise shifting environments:

[Having] others who are going through the same thing as you. That whole “Do you feel the same way I do?”—and often, someone does. It reassures you that […] you’re not alone. And sometimes, it just makes things a bit easier when you’re part of something where others feel the same. Or if someone has good input, tips, and tricks on how to handle things differently. (Group interview 2, GP trainee 6)

Beyond immediate hospital GP peers, trainees also valued the wider GP trainee network as a community for sharing experiences, strategies, and advice on managing frequent transitions. These peer communities offered a sense of belonging that hospital departments rarely provided, helping sustain belonging, and linking the experience of repeated transition to the broader question of learning as a GP trainee in hospital settings.

### Working and learning at the margins

The final theme describes how GP trainees’ everyday participation within hospital departments was shaped by a dual position: they were both learners and service providers, positioned at the margins of hospital communities yet expected to contribute substantially to clinical work. Two aspects illustrate this dynamic: 1) Fitting in and finding a place; and 2) Balancing educational needs with service demands.

#### Fitting in and having a place

GP trainees’ learning depended greatly on how they were received and integrated into hospital teams. When training outside their own specialty, GP trainees described becoming members of the departmental community as both necessary and sometimes difficult. Many recounted positive experiences of inclusion, emphasising friendly colleagues who learned their names, recognised their competencies, and involved them in relevant tasks:

They quickly learn your name […]. They know who people are. I think that means quite a lot for my well-being – or, you could say, my job satisfaction – being here, that you’re not just someone passing through. (Group interview 6, GP trainee 24)

Such efforts helped GP trainees feel part of the team, enhancing their sense of belonging, and supported their professional wellbeing. However, these experiences varied, and some departments were described as disinterested or even exclusionary:

For me, […] it was clear that, “You are just GP doctors, you just need to get through”. “We don’t value you much. Just fill the gaps”. (Group interview 9, GP trainee 39)

In such cases, GP trainees felt more like temporary visitors than members of the workplace community. Belonging, they explained, was rarely automatic but required active effort from both colleagues and themselves:

As a GP trainee, sometimes you can get a bit forgotten in the group. So, if there’s something you really want, in terms of learning for the GP role, you have to speak up for yourself. You must demand it, or say it, or make people aware of it. You can easily just become one of the groups, which then again is not the group you actually fit into. (Group interview 1, GP trainee 1)

Even where intentions were positive, GP trainees still sensed that they were not fully prioritised, especially when educational activities were designed primarily for hospital specialty trainees. GP trainees reflected that this lack of prioritisation stemmed from the fact that they had already chosen another field. As some noted, departments often focused their educational resources on potential future colleagues within their specialty. This was also evident in our observations. For example, conference rooms displayed boards tracking the competency progression of other trainees, while no equivalent overview existed for GP trainees. Yet, GP trainees argued that their learning should still matter: understanding hospital systems and patient pathways was central to effective general practice, and ultimately, also beneficial to the wider healthcare system:

A part of me hopes that someone has actually considered that “these trainees are going to work in general practice afterwards – that what we do here can be useful there”. […] I hope there’s someone who’s thought, “We want to educate good GPs” because in the long run that will help reduce the hospital workload. (Group interview 3, GP trainee 12)

#### The challenge of balancing educational needs and service demands

Across group interviews, GP trainees expressed concern that the short duration of rotations combined with high service demands left their learning needs overlooked. Several felt valued mainly for their labour, describing themselves as “just a pair of hands” or “cheap labour”, or there to “plug gaps in the rota”. Others spoke of being “steamrolled” by the pace of work, leaving little room to assert their educational needs:

There will be times when we find ourselves thinking, “What the hell am I doing here?” […] You just become part of the system, keeping things running. (Group interview 4, GP trainee 16)

We are here largely for the sake of the hospital system. […] First and foremost, we are here because there is a huge workload that we are expected to help manage. And education comes second. And sometimes third, fourth. […] It’s not about, “Let’s design the best possible training programme for future GPs”. (Group interview 7, GP trainee 27)

While many appreciated efforts done to support their education, trainees found that service pressures often dictated shift allocation, task distribution, and responsibility levels:

It is completely unreasonable, the amount of… well, responsibility. It is just expected. We come from general practice, and then […] suddenly, we are expected to [coordinate flow and allocation]. after just half a day of training […] for an entire night shift. I think that is completely unrealistic. (Group interview 3, GP trainee 10)

These experiences illustrate a persistent tension between service and education. For some trainees, the deprioritisation of learning made it difficult to discern the purpose of hospital rotations and raised questions about the coherence of their training trajectory.

## Discussion

This study explored how GP trainee experience training outside their own specialty and how they make sense of learning, relevance, and belonging while “learning away from home”. A key strength of the study is the combination of field observations and group interviews, which enabled us to examine both how learning and collaboration unfolded in everyday practice, and how trainees retrospectively made sense of their experiences. This methodological integration strengthens our analysis, which shows that GP trainees continuously negotiated meaning, learning, and belonging while situated in hospital settings.

Hospital rotations were described as valuable opportunities to gain exposure to different departments, specialist expertise, and a concentrated selection of patients, supporting the development of their clinical competence relevant to future GP practice. At the same time, these opportunities coexisted with notable tensions, particularly in finding meaning in these rotations, adapting to frequent transitions and in working and learning at the margins.

A major theme in this study concerns how GP trainees sought meaning and relevance when training outside their home specialty. Although hospital rotations are formally intended to support GP-relevant skill development, GP trainees often expressed ambivalence about their learning. This ambivalence reflects a tension between exposure to specialist medicine and uncertainty about how such experiences translate into future GP practice. Similar tensions have been described in previous research on GP training in hospital settings [1,2,6], where relevance is not inherent in the clinical encounter but must be actively constructed.

Interpreted in this light, trainees’ frustration with tasks perceived as irrelevant should not be understood simply as dissatisfaction, but as an expression of misalignment between educational intentions and organisational realities, a phenomenon well-documented in postgraduate medical training [23,24,25]. Our findings extend this literature by showing how short and frequent rotations may intensify this tension. Other studies have similarly highlighted compromised educational quality for GP trainees, including limited protected time and weak alignment between hospital activities and GP competencies [2,25,26].

A key contribution of this study is showing that perceptions of relevance were not fixed but actively negotiated. The diversity of views among GP trainees indicates that relevance is shaped less by the clinical encounter itself than by the learner’s perspective. This insight adds nuance to earlier work by drawing attention to the interpretive process through which trainees actively construct meaning and relevance in hospital settings. From an educational perspective, this underscores the importance of supervisory practices that explicitly connect hospital work to GP learning objectives and create space for reflection on transferability.

Belonging emerged as another key dimension shaping learning during hospital rotations. While many described supportive colleagues, experiences varied considerably, echoing findings from previous research. Some felt welcomed and included [26,27], while others felt like temporary staff, “just another GP trainee” filling rota gaps with limited attention to their learning needs [1,2,13,28]. These variations suggest that experiences of inclusion are not uniform but depend heavily on local departmental culture and supervisors’ understanding of the GP role.

Frequent transitions played a critical role in shaping these experiences of belonging. While transitions are a common feature of medical training [29], GP trainees encounter them repeatedly due to the structure of their specialist training. Multiple short rotations across different specialties mean that disorientation is not a temporary stage but a recurrent condition, and often without the stabilising sense of progression available to hospital-based trainees. In this way, continuity itself becomes an educational challenge, shaping GP trainees’ sense of belonging and professional development.

Drawing on Lave and Wenger’s concept of legitimate peripheral participation [30], we interpret these findings as indicating a disrupted learning trajectory [29]. Rather than gradually moving from peripheral to full participation within a community of practice, GP trainees were left in a state of recurring peripherality as they re-entered new environments without time to build relationships or consolidate learning. Importantly, this peripheral positioning was not limited to hospital rotations. Because GP trainees spend extended periods away from general practice, they also described feeling peripheral when returning to general practice, having to re-establish routines, networks, and professional footing after years in hospital settings. We conceptualise this as a form of “double peripherality”, in which the experience of peripherality becomes a recurring feature of their professional journey rather than a temporary stage of learning.

Finally, our findings highlight how GP trainees compensated their sense of peripherality by forming peer-based communities, which provided emotional support and practical guidance during transitions. Through these relationships, trainees created alternative spaces for participation that softened some of the isolation caused by repeated peripherality. Attending to these social and relational dimensions of learning may be as important as ensuring curricular relevance, particularly for trainees “learning away from home”.

## Conclusion

This study explored GP trainees’ experiences of hospital-based training, identifying both opportunities for learning and challenges related to relevance, belonging and continuity. GP trainees’ experiences were shaped by how tasks and learning were framed, as well as by the management of frequent transitions across clinical settings. Our analysis highlighted three overarching themes: Finding meaning away from home, learning in frequent transitions, and working and learning at the margins. Together, these themes revealed how GP trainees often occupied a form of double peripherality, positioned at the edges of hospital teams while also spending extended periods away from their base in general practice. Planning, supervision, and peer support emerged as critical in enabling learning experiences and supporting trainees’ sense of professional identity. These findings underscore the importance of structuring hospital rotations to support not only educational value and continuity, but also the development of professional communities that foster relevance, belonging and professional identity formation for GP trainees.

## Limitations

Our findings are situated within the Danish medical education model, where GP training includes multiple short hospital rotations, with only limited and infrequent return to general practice settings (typically one day per month) during this phase of training. While transferability to other contexts must be considered carefully, we have aimed to provide a rich description of the organisational settings shaping trainees’ experiences. Such contextual detail may support readers in judging the relevance of the findings to systems with similar structures or transitional demands.

Another limitation of this study concerns the potential for selection bias, as participation in both observations and group interviews was voluntary. It is therefore possible that trainees with particularly strong views (positive or negative) were more inclined to participate. However, across observations and interviews we encountered a wide range of perspectives, including contrasting experiences and nuanced reflections, suggesting that the data captured substantial variation in how trainees navigated hospital-based training.

## Data Accessibility Statement

The data from the study are not publicly available due to ethical considerations regarding participants’ anonymity, but they are available from the corresponding author upon reasonable request. The data are in Danish, and only an anonymised extract will be provided.

## Appendix

**Appendix 1:** Interview guide, GP trainees.

THEMES	QUESTIONS

Introduction to the project and interviewer	The group interview concerns your experiences of the hospital rotation, which is part of your specialist training in general practice. It forms part of a research project exploring GP trainees’ hospital-based training. In addition to interviews with GP trainees, we have conducted field observations. Participation is voluntary. You will be pseudonymised in all written material. It is also important to emphasise that what is said here stays within this group — please do not discuss what others have said outside the interview. The interview will be audio-recorded and transcribed, and the interviewer will take notes during the session. It will last approximately one hour. I have prepared an interview guide in advance. Please remember that there are no “right” or “wrong” answers — I am interested in your individual perspectives and in bringing multiple viewpoints into the discussion. You do not have to agree with each other. Any questions before we begin?

Participant introduction	Please begin by briefly introducing yourself: your name, your current hospital department, and a short outline of your training so far.

Initial reflections	To begin with: If you were to tell a new GP trainee about the hospital rotation, what would you tell them?

Before the hospital rotation – expectations	What expectations did you have before starting your hospital training? Why? What stories or experiences had you heard from others before you started? For example, are there particular reputations or shared “stories” about certain departments? (Encourage interaction: “Does that sound familiar to the rest of you?”/“Would anyone see it differently?”)

During the hospital rotation	From your perspective: What is the main purpose of having a hospital rotation as part of GP specialist training? What, for you, makes a hospital rotation a good learning experience? *Can you give examples of departments that succeed, and perhaps some that do not?* Are there situations where you are assigned a different role or function than other trainees in the department? *In clinical work or meetings?* Are there functions where your generalist skills as a GP trainee are particularly used or valued? In what ways do you think GP trainees contribute to hospital departments? *Can you provide examples of departments that successfully integrate GP trainees?* We know from previous research that the transitions between departments can be challenging. *How have you experienced these? What makes them difficult or manageable?*

Workplace community	How do you experience being part of the hospital team as a GP trainee? How do you think others in the department see you? Is it ever explicitly mentioned that you are GP trainees? How do you see your own role? Do you feel that your learning is prioritised on an equal level with other trainees?Can you think of examples where it has — or has not — been the case? (Encourage reflection: “Do others recognise this experience?”/“Is that similar across departments?”)

Learning potential	Overall, what do you think you learn most from your hospital rotation? What experiences do you think will be most useful in your future GP practice?

Closing reflections	Apart from what we have discussed, is there anything you think could be improved in the hospital rotation? Is there anything important that we have not talked about but that you think I should know?
